# Protective role of Bacillus Calmette–Guérin vaccine in Alzheimer's disease progression: A systematic review and meta-analysis

**DOI:** 10.1016/j.heliyon.2024.e27425

**Published:** 2024-03-06

**Authors:** Tungki Pratama Umar, Nityanand Jain, Bella Stevanny, Binish Javed, Andyra Priandhana, Reynold Siburian, Andrejs Kostiks

**Affiliations:** a Division of Surgery and Interventional Science, Faculty of Medicine, University College London, London, UK; b Faculty of Medicine, Riga Stradinš University, Riga, LV 1007, Latvia; c Faculty of Medicine, Universitas Sriwijaya, Palembang, Indonesia; d Atal Bihari Vajpayee Institute of Medical Sciences and Dr. Ram Manohar Lohia Hospital, New Delhi, India; e Jakarta Heart Center, Jakarta, Indonesia; f Department of Neurology, Riga East Clinical University Hospital, Riga, Latvia

**Keywords:** BCG vaccine, Alzheimer's disease, Prevention, Dementia, Bladder cancer

## Abstract

Introduction: Alzheimer's disease (AD) represents a significant societal challenge, highlighting the need to explore effective prevention and treatment approaches. Recent literature has suggested that Bacillus Calmette-Guérin (BCG) vaccine may be a viable therapeutic option for immune disorders associated with AD due to its immunomodulatory properties and protection against various diseases.

**Methods:**

This systematic review aimed to evaluate the association of BCG vaccine in the prevention of AD using six medical-scientific databases. A meta-analytical approach was undertaken to estimate the risk of AD incidence in patients with and without BCG vaccine exposure, followed by subgroup analyses. A risk of bias (RoB) assessment was performed using the Newcastle-Ottawa Scale (NOS).

**Results:**

Six cohort studies meeting our inclusion criteria were included (47,947 participants) in the study. From our meta-analysis, intravesical BCG vaccine administration lowered the risk of incidence of AD by 26% in non-muscle-invasive bladder cancer (p < 0.00001). Subgroup analyses showed that BCG vaccination showed a potentially notable preventive effect on AD in older adults (>75 years) and female participants. Conversely, significant heterogeneity in results was observed among male participants and those aged <75 years. The RoB was low in three studies and unclear in the remaining studies.

**Conclusions:**

Although our results support the potential benefits of BCG vaccine in preventing AD in specific demographics, we remain cautious about interpreting such results. Further research examining the implications of BCG vaccination for prevention and possible treatment of AD should be undertaken in the future.

## 1 Introduction

Alzheimer's disease (AD) is a prominent cause of dementia in older adults and ranks fifth among the leading causes of death globally. In 2019, AD affected approximately 57.4 million individuals worldwide. Due to a rapidly aging population, it is projected that the number of individuals affected by AD may nearly triple by the year 2050 [1]. Furthermore, AD imposes a significant burden on individuals, their families, healthcare systems, and society in general [2]. Given the intricacy of AD and its underlying mechanisms, which renders the condition clinically challenging to manage, especially during the disease's advanced stages, it is crucial to explore efficacious methods for forestalling or altering the course of progression of AD [3].

Multiple factors, such as genetics, the environment, and lifestyle contribute in the development and progression of AD [3]. Researchers are constantly analysing various methods, such as pharmacological treatments, dietary adjustments, and immunomodulatory therapy [4,5], to delay or prevent the onset of AD and enhance treatment outcomes. A proposed novel method for treating immune disorders associated with AD involves utilizing Mycobacterium bovis-derived Bacillus Calmette-Guérin (BCG) vaccine [6]. The BCG vaccine has been used to prevent tuberculosis (TB) and other mycobacterial illnesses, particularly in TB-endemic regions, and is considered one of the most administered vaccines. Additionally, it is frequently included in infant vaccination schedules [7].

The effect of BCG vaccination on regulatory T cells (Tregs) and cytokine interleukin-10 (IL-10) has been extensively researched from an immune regulation standpoint [8]. Besides, evidence indicates that the BCG vaccine may offer protection against diseases like leprosy, non-tuberculous mycobacteria (NTM) and respiratory virus infections [9,10]. Additionally, countries with mandatory BCG vaccination have demonstrated lower rates of coronavirus disease 2019 (COVID-19) transmission and fatalities in contrast to those without this requirement [11]. Currently, research on the possible AD preventative applications of the BCG vaccine is increasing, as immunological dysfunction and neuroinflammation have been known to contribute to AD's aetiology. The immunomodulatory properties of the BCG vaccine and its potential to induce trained immunity suggest that it may alter the progression of AD or delay its onset [12,13].

A study found that BCG vaccination reduced hippocampal dendritic spine pathology, possibly due to changes in dendritic arborization and spine shape or increased expression of synuclein (α-SYN) and postsynaptic density protein 95 (PSD-95). The mechanisms that lead to elevated interferon gamma (IFN-γ) or IL-4/JAK2/STAT3 levels following BCG administration may also be significant [14].

Hence, we aimed to evaluate the current literature on the association between BCG vaccination and AD prevention. The results of this study could significantly impact the design of the next treatment trials and the development of preventive approaches to reduce AD prevalence worldwide. Furthermore, it could illuminate the broader potential of BCG vaccination as a prophylactic measure against diseases associated with immune dysregulation.

## 2 Methods

We undertook a systematic literature review across several biomedical databases to collect and analyze relevant publications that reported on the effects of BCG vaccination in preventing the onset or delay the progression of AD. We adhered to the Preferred Reporting Items for Systematic Reviews and Meta-Analysis (PRISMA) 2020 statement guideline [15]. The review procedure has been registered prior to data extraction in the International Prospective Register of Systematic Reviews (PROSPERO; CRD42023432580) database.

### 2.1 Inclusion and exclusion criteria

We focused on scientific literature that reported about the effects of BCG vaccination on the development of AD in the later stages of patients’ life. Observational studies reporting on adult participants aged over 18 years were considered. The studies were required to have used a standardized examination method. The use of BCG vaccine could have been in the form of routine vaccination at childhood or as an immunotherapy. The included studies were required to study the effects using both exposed (got BCG vaccine as mandatory vaccination or therapeutic purpose for disease other than dementia) and non-exposed (BCG-naïve/never received BCG) groups. We excluded protocols, case reports, conference abstracts, literature reviews, case series, opinion pieces, and unretrievable full texts. To maintain data comparability, only full text publications in English were included in our systematic review.

### 2.2 Search strategy

A comprehensive literature search was conducted across six biomedical databases – Cochrane Library, EbscoHost, Epistemonikos, Google Scholar, PubMed, and Scopus. The search was performed in July 2023 with no time limitations placed for included studies. Following the literature search, the studies underwent title and abstract screening, and only studies in concordance with the inclusion criteria were evaluated for further screening. Duplicate records were also removed at this stage. The search string used in the present study is presented in Table 1.

**Table 1:** Search string.

Biomedical Database	Search String Query	Number of publications
**EbscoHost**	(“Bacillus Calmette-Guérin” OR “BCG” OR “BCG vaccine” OR “Tuberculosis vaccine” OR “TB vaccine”) AND (“Alzheimer's disease” OR “AD” OR “Alzheimer's dementia” OR “Dementia” OR “cognitive disorder” OR “neurodegenerative disorder”)	3
**Epistemonikos**	(“Bacillus Calmette-Guérin” OR “BCG” OR “BCG vaccine” OR “Tuberculosis vaccine” OR “TB vaccine”) AND (“Alzheimer's disease” OR “AD” OR “Alzheimer's dementia” OR “Dementia” OR “cognitive disorder” OR “neurodegenerative disorder”)	77
**Cochrane Library**	(“Bacillus Calmette-Guérin” OR “BCG” OR “BCG vaccine” OR “Tuberculosis vaccine” OR “TB vaccine”) AND (“Alzheimer's disease” OR “AD” OR “Alzheimer's dementia” OR “Dementia” OR “cognitive disorder” OR “neurodegenerative disorder”)	75
**PubMed**	(“Bacillus Calmette-Guérin” OR “BCG” OR “BCG vaccine” OR “Tuberculosis vaccine” OR “TB vaccine”) AND (“Alzheimer's disease” OR “AD” OR “Alzheimer's dementia” OR “Dementia” OR “cognitive disorder” OR “neurodegenerative disorder”)	146
**Scopus**	(“Bacillus Calmette-Guérin” OR “BCG” OR “BCG vaccine” OR “Tuberculosis vaccine” OR “TB vaccine”) AND (“Alzheimer's disease” OR “AD” OR “Alzheimer's dementia” OR “Dementia” OR “cognitive disorder” OR “neurodegenerative disorder”)	263
**Google Scholar**	“Bacillus Calmette-Guérin”, “BCG”, “Alzheimer's disease”, “Dementia”	223

### 2.3 Study selection

Two reviewers (BS and AP) independently used Rayyan for initial title and abstract screening [16]. Reviewer discrepancies were resolved by re-evaluating the text against the inclusion criteria and discussion among other study authors. Subsequently, full published texts of all relevant included publications were collected and re-assessed by two independent reviewers (RS and TPU). The researcher conflicts were settled similarly during the full-text screening phase. If no settlement could be actualized, two moderators (NJ and AK) were present to assess the discrepancies in consultation with input from other authors.

### 2.4 Data extraction and risk of bias appraisal

Two authors (BJ and AP) independently gathered data from the included studies. The data variables that were extracted included – author list, study design, country, population, study period, age, male/female proportion, BCG administration, patient population type, AD proportion, follow-up period, and BCG dosing. Two other reviewers (TPU and BJ) independently appraised the risk of bias (RoB), with discrepancies resolved via discussion among the study authors. Methodological quality of the selected studies was assessed using the Newcastle-Ottawa Scale (NOS) scale, which consists of three sections: selection, comparability, and outcome. Each study is assessed using a point system across three dimensions and categorised as low (7–9 points), moderate (4–6 points), or high (0–3 points) RoB [17]. The modified NOS scale was used for cross-sectional studies. The studies were classified as low (7–8 points), moderate (5–6 points), and high (0–4 points) RoB [18].

### 2.5 Data analysis

The extracted data was recorded in Microsoft Excel 365. For the meta-analysis, study heterogeneity was established using the Higgins I^2^ statistic. A p-value of <0.100 and I^2^ of >50% indicates considerable heterogeneity [19]. Meta-analyses were conducted using Review Manager (RevMan) Version 5.4.1, employing both random-effects and fixed-effects models. Forest plots with 95% confidence interval (CI) were used for visualization. Meta-analysis was done for AD incidence that was reported in the included studies. We also conducted subgroup analysis based on gender and age groups. Data is presented as Odd's ratio.

## 3 Results

### 3.1 Study characteristics

**Fig. 1:**
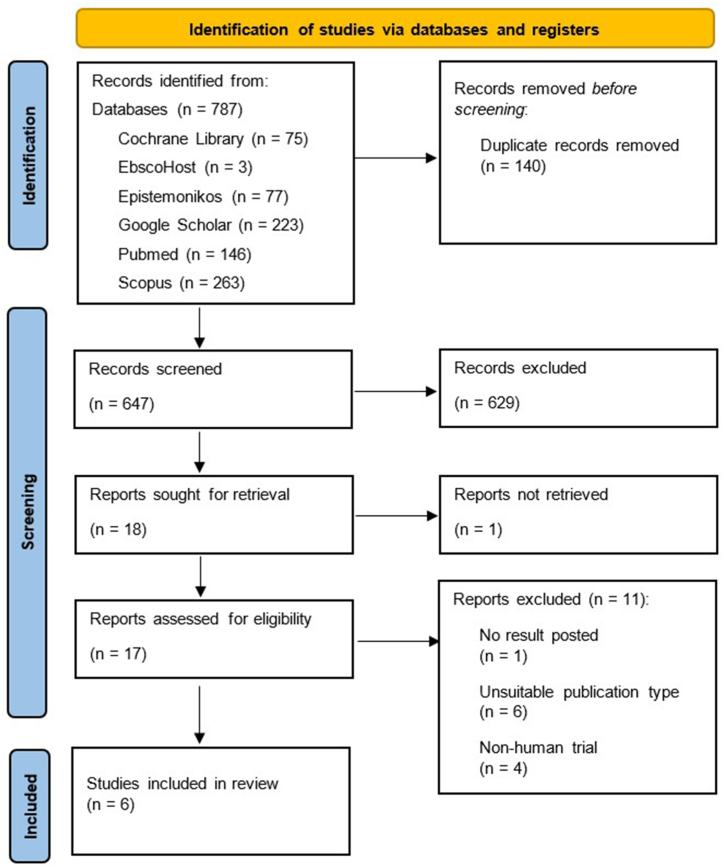
PRISMA flowchart.

Our search retrieved 787 records, 140 of which were duplicate records and removed (Fig. 1). Further 629 were excluded following title and abstract screening. One report was excluded due to the irretrievable full text. The remaining 11 records were excluded due to unsuitable publication type (n = 5), no result posted (n = 1), non-human trial (n = 4), and abstract only (n = 1), resulting in the inclusion of six cohort studies [[20], [21], [22], [23], [24], [25]]. All included studies followed retrospective study design. Among the included studies, one was multi-centered [23] and the remaining were single-centered cohorts. Five of the included studies were conducted in USA [20,[22], [23], [24], [25]], while two studies were conducted in Israel [21,23] (Table 2).

**Table 2:** Study characteristics.

Author	Study Design	Country	Population	Study Period	Mean Age (BCG)	Male/Female (BCG)	BCG Administration	Patient population	AD proportion	Follow -up period	BCG dosing
Dow et al. (2022) [20]	Prospective cohort	USA	49 (BCG only)	N/A	64.3 ± 6.7	21/28	Intradermal (vaccination)	BCG naïve, immunocompetent, with a family history of dementia	34 low risk, 5 intermediate risk, 10 high risk	9 months	0.1 mL (2 × 10^5^ CFU)
Gofrit et al. (2019) [21]	Retrospective cohort	Israel	1372 (878 BCG, 494 non-BCG)	1966 to 2018^a^	67.5 ± 12.6	730/148	Intravesical (immuno-therapy)	BC	21/878 (2.4%) BCG, 44/494 (8.9%) non-BCG	Median: 8 years	12.5 mg per vial containing 2–8 x 10^8^ CFU
Kim et al. (2021) [22]	Retrospective cohort	USA	1290 (319 BCG, 971 non-BCG)	1984 to 2020^a^	69 ± 11.9	237/82	intravesical (immuno-therapy)	NMIBC	2/16 (BCG), 26/103 (non- BCG)	Median: 3 years (IQR, 1–6 years)	N/A
Klinger et al. (2021) [23]	Retrospective cohort	Israel, USA	12,185 (2301 BCG, 9884 non-BCG)	2000 to 2020^a^	N/A	CHS: 1341/237; HUH: 350/58	Intravesical (immuno-therapy)	BC	CHS: 75/1578 (4.8%); HUH: 13/408 (3.2%)	3.5–7 years	N/A (≥3 instillations within a 120-day period)
Makrakis et al. (2022) [24]	Retrospective cohort	USA	26,584 (13,496 BCG, 13,088 non-BCG)	2004 to 2015^a^	76.7 ± 6.6	10,708/2788	Intravesical (immuno-therapy)	High-risk NMIBC	964/2192 (BCG), 1228/2192 (non BCG)	Median: 39 months	N/A (≤6 doses to 13+ doses)
Weinberg et al. (2023) [25]	Retrospective cohort	USA	6467 (3388 BCG, 3079 non-BCG)	April 18, 2021 to March 28, 2023	69.9 ± 9.3	2605/783	intravesical (immuno-therapy)	NMIBC	202/3388 (BCG), 262/3079 (non-BCG)	15 years	N/A

a Database period, BC = bladder cancer, BCG = Bacillus Calmette–Guérin, CHS = Clalit Health Services; CFU = colony-forming unit, HUH = Hadassah University Hospitals; NMIBC = nonmuscle-invasive bladder cancer, N/A = not available.

### 3.2 Meta-analysis

**Fig. 2:**
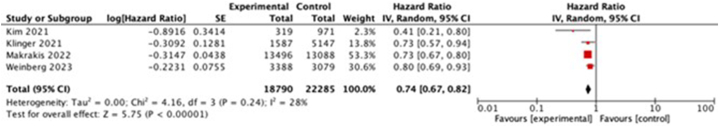
Pooled unadjusted meta-analysis showing the association between intravesical BCG administration and incidence of AD. CI, confidence interval.

**Fig. 3:**
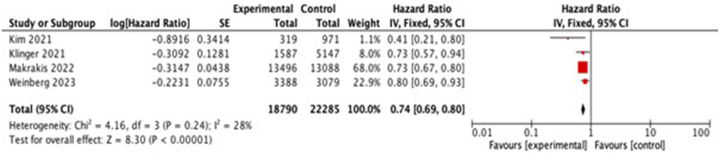
Pooled adjusted meta-analysis showing the association between intravesical BCG administration and incidence of AD. CI, confidence interval.

Two studies were excluded from meta-analysis since one study assessed different parameters (APP score) [20], and one study had different presentation of HR values [21]. This left us with four studies that were appropriate for inclusion in the meta-analysis. We observed that intravesical BCG administration lowered the risk of AD incidence by 26% (HR 0.74; 95% CI = 0.67–0.82, p < 0.00001, I^2^ = 28%, Fig. 2) in non-muscle-invasive bladder cancer for unadjusted data. Nonetheless, these findings were found to be consistent with our findings on the pooled adjusted (for age and gender) effects (HR 0.74; 95% CI = 0.69–0.80, p < 0.00001, I^2^ = 28%; Fig. 3).

**Fig. 4:**
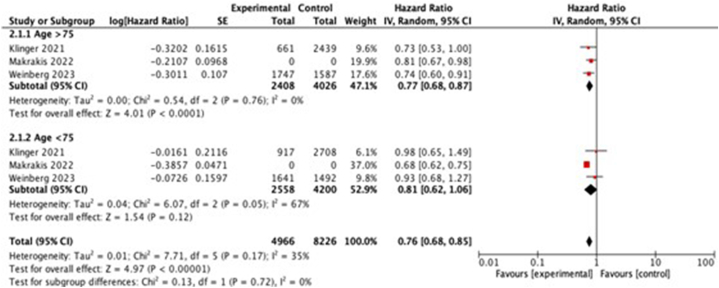
Subgroup analysis (based on age) investigating the association between intravesical BCG administration and incidence of AD. CI, confidence interval.

**Fig. 5:**
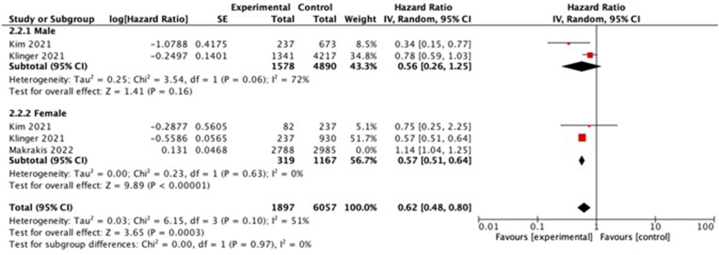
Subgroup analysis (based on gender) investigating the association between intravesical BCG administration and incidence of AD. CI, confidence interval.

Subgroup analysis revealed substantial heterogeneity among specific age groups (age <75 years) (Fig. 4) and sex (male participants) (Fig. 5). We found that the impact of BCG vaccine administration as an immunotherapy of bladder cancer is beneficial to prevent AD development in people aged >75 years. However, the same observation was not noted in the younger age group (<75 years). Furthermore, the impact of BCG vaccine to prevent AD development is only observed in female group.

We were unable to perform publication bias assessment due to study paucity (n < 10) and small sample sizes. Hence, we advise that these findings should be interpreted with caution.

### 3.3 Risk of Bias

**Fig. 6:**
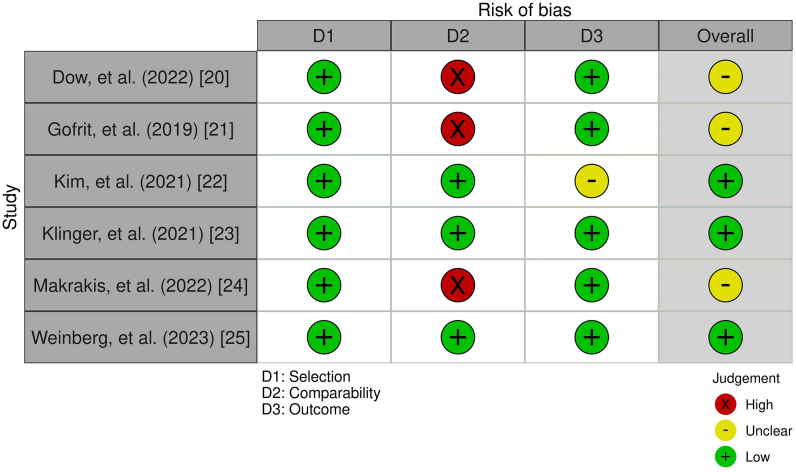
Risk of Bias assessment.

Risk of bias assessment showed low risk for three studies [22,23,25] and unclear risk for three other studies [20,21,24] (Fig. 6). All studies had unclear adequacy of follow-up time, one study have short follow-up period that might not be long enough for the outcome to occur [22], and three studies had unclear comparability of cohorts due to issues in study design or analysis [20,21,24]. All included studies demonstrated that the primary outcome was absent at baseline. Furthermore, the studies couldn't sufficiently address the impact of confounders such as age and gender. Since most of the included studies were retrospective in nature, they were predisposed to certain selection bias.

## 4 Discussion

Amyloidogenic protein accumulation is the hallmark of AD, with literature suggesting that neuroinflammation may be primarily instigated by microglia [26]. Additionally, immunosuppression mediated by Foxp3+ regulatory T-cells has been implicated in the development of AD. It has been shown that an increase in number of CD8^+^ T effector memory CD45RA + cells in the peripheral blood and cerebrospinal fluid could potentially serve as a pathognomonic immune marker in AD [27]. Current understanding of AD prevention remains limited. Immunotherapy, including the use of BCG vaccine, is being employed or tested to delay AD development.

The concept of utilizing the BCG vaccine as a preventive measure for AD is grounded in a single epidemiological observation, which identified a negative correlation between AD and BCG vaccination, particularly in populations where BCG vaccination is mandatory [21]. A recent comprehensive study based on Surveillance, Epidemiology, and End Results (SEER) inferred that the patients treated with BCG exhibited improved disease-specific survival rates, with even greater overall survival rates [21]. The exact mechanisms underlying the intravesical administration of BCG vaccine remain unclear. However, it is noteworthy that this type of administration results in a significant increase in IL-2 levels, believed to contribute to the expansion of neuroprotective regulatory T cells.

Research suggests that in animal models of AD and Japanese encephalitis, the BCG vaccine may reduce neuroinflammation by recruiting anti-inflammatory monocytes to the brain [22,23]. Furthermore, studies conducted on mouse models of AD have suggested that administration of the BCG vaccine results in a reduction of cognitive deficits. These findings correlate with a notably high influx of CD45, interleukin-10-secreting monocytes detected within the choroid plexus and perivascular spaces. Additionally, there are increased neuronal dendritic complexity and higher levels of postsynaptic density-related proteins, along with a shift towards an anti-inflammatory state in brain cytokines and an elevation in brain-derived neurotrophic factor [28]. Another hypothesis suggests that BCG vaccine may have a non-specific activating effect on the innate and adaptive immune systems, thereby enhancing the ability of microglial cells to clear up Aβ [20].

The BCG vaccination showed a potentially notable preventive effect on AD in older adults (75+ years) and female populations. The lack of information available beyond a 5- or 10-year follow-up may establish the vaccine's stronger influence, with earlier BCG vaccination potentially tied to a lower risk of AD-related mortality [25]. The higher AD incidence rate among females may be attributed to their relatively lower vaccination rates compared to males [29]; however, this remains inconclusive [21].

This study represents the meta-analyses examining the association between intravesical BCG vaccine and AD incidence. Our findings contribute to the existing research on the potential advantages of intravesical BCG vaccine. Our analysis, however, also highlights several limitations. Specifically, our inclusion criteria were limited to peer-reviewed studies published in English, and this may have introduced language and publication bias. Additionally, the scarcity of studies included in our analysis may limit the generalizability of our findings. Potential influences of patient characteristics, such as sex and comorbidities, were unable to be evaluated in our study. However, we conducted a thorough search across multiple databases with rigorous inclusion and exclusion criteria to ensure the strength of our research. Moreover, all included studies were conducted in nations without a mandated BCG vaccination schedule for children, which could indicate that the findings cannot be applied to nations where BCG vaccination is a requirement. Furthermore, there may be survival bias due to different cancer stages that are not thoroughly characterized in the analysis (just the ultimate information on AD cases).

Our study demonstrated some levels of statistical heterogeneity, which may have arisen from variables such as differences in follow-up time, mean age, gender, and comorbidities. Subgroup analysis showed a significant heterogeneity on male gender and participants aged <75 years. There is a need for high-quality prospective research to assess the association between BCG and Alzheimer's incidence. BCG has a long history of use and a favourable safety profile, which makes it a promising candidate for further investigation.

## 5 Conclusions

Our results indicate that intravesical BCG vaccine is associated with decreased AD development risk in bladder cancer patients. This finding supports potential benefit of BCG vaccine as preventative measure of AD although further studies are still warranted, particularly for the possibility of treatment purpose (where there is no evidence available till the end of this systematic review writing).

## Ethics approval

Not applicable.

## Funding

None.

## Data availability statement

Data Available upon request from the corresponding author.

## CRediT authorship contribution statement

**Tungki Pratama Umar:** Writing – review & editing, Writing – original draft, Visualization, Software, Resources, Project administration, Methodology, Investigation, Formal analysis, Data curation, Conceptualization. **Nityanand Jain:** Writing – review & editing, Validation, Resources, Project administration, Methodology, Investigation. **Bella Stevanny:** Writing – original draft, Visualization, Software, Investigation, Formal analysis, Data curation. **Binish Javed:** Writing – original draft, Investigation, Data curation. **Andyra Priandhana:** Writing – original draft, Investigation, Data curation. **Reynold Siburian:** Writing – original draft, Investigation, Data curation. **Andrejs Kostiks:** Writing – review & editing, Validation, Supervision, Project administration, Methodology.

## Declaration of competing interest

The authors declare that they have no known competing financial interests or personal relationships that could have appeared to influence the work reported in this paper.

## References

[1] Nichols E., Steinmetz J.D., Vollset S.E., Fukutaki K., Chalek J., Abd-Allah F. (2022). Estimation of the global prevalence of dementia in 2019 and forecasted prevalence in 2050: an analysis for the Global Burden of Disease Study 2019. Lancet Public Health.

[2] Aranda M.P., Kremer I.N., Hinton L., Zissimopoulos J., Whitmer R.A., Hummel C.H. (2021). Impact of dementia: Health disparities, population trends, care interventions, and economic costs. J. Am. Geriatr. Soc..

[3] Breijyeh Z., Karaman R. (2020). Comprehensive review on Alzheimer's disease: causes and treatment. Molecules.

[4] Cummings J., Lee G., Nahed P., Kambar M.E.Z.N., Zhong K., Fonseca J. (2022). Alzheimer's disease drug development pipeline: 2022. Alzheimer's Dementia.

[5] Wilson D., Peters R., Ritchie K., Ritchie C.W. (2011). Latest advances on interventions that may prevent, delay or ameliorate dementia. Ther Adv Chronic Dis.

[6] Adesanya O.A., Uche-Orji C.I., Adedeji Y.A., Joshua J.I., Adesola A.A., Chukwudike C.J. (2020). Expanded scope of Bacillus calmette-guerin (BCG) vaccine applicability in disease prophylaxis, diagnostics, and immunotherapeutics. Infect Microbes Dis.

[7] Fritschi N., Curtis N., Ritz N. (2020). Bacille Calmette Guérin (BCG) and new TB vaccines: specific, cross-mycobacterial and off-target effects. Paediatr. Respir. Rev..

[8] Xu H., Jia Y., Li Y., Wei C., Wang W., Guo R. (2019). IL-10 dampens the Th1 and Tc activation through modulating DC functions in BCG vaccination. Mediat. Inflamm..

[9] Orujyan D., Narinyan W., Rangarajan S., Rangchaikul P., Prasad C., Saviola B. (2022). Protective efficacy of BCG vaccine against Mycobacterium leprae and non-tuberculous mycobacterial infections. Vaccines.

[10] Yitbarek K., Abraham G., Girma T., Tilahun T., Woldie M. (2020). The effect of Bacillus Calmette-Guérin (BCG) vaccination in preventing severe infectious respiratory diseases other than TB: implications for the COVID-19 pandemic. Vaccine.

[11] Irfani T.H., Siburian R., Nabila R., Umar T.P. (2020). Tuberculosis and coronavirus disease 2019 (COVID-19) from A clinical perspective: a systematic review. Medeni Med. J..

[12] Covián C., Fernández-Fierro A., Retamal-Díaz A., Díaz F.E., Vasquez A.E., Lay M.K. (2019). BCG-induced cross-protection and development of trained immunity: implication for vaccine design. Front. Immunol..

[13] Klein B.Y., Greenblatt C.L., Gofrit O.N., Bercovier H. (2022). Bacillus calmette–Guérin in immuno-regulation of alzheimer's disease. Front. Aging Neurosci..

[14] Li Q., Wang X., Wang Z.H., Lin Z., Yang J., Chen J. (2022). Changes in dendritic complexity and spine morphology following BCG immunization in APP/PS1 mice. Hum. Vaccines Immunother..

[15] Page M.J., McKenzie J.E., Bossuyt P.M., Boutron I., Hoffmann T.C., Mulrow C.D. (2021). The PRISMA 2020 statement: an updated guideline for reporting systematic reviews. BMJ.

[16] Ouzzani M., Hammady H., Fedorowicz Z., Elmagarmid A. (2016). Rayyan—a web and mobile app for systematic reviews. Syst. Rev..

[17] Luchini C., Stubbs B., Solmi M., Veronese N. (2017). Assessing the quality of studies in meta-analyses: advantages and limitations of the Newcastle Ottawa Scale. World J. Meta-Anal..

[18] Liana P., Liberty I.A., Murti K., Hafy Z., Salim E.M., Zulkarnain M. (2022). A systematic review on neutrophil extracellular traps and its prognostication role in COVID-19 patients. Immunol. Res..

[19] Higgins J.P.T., Thompson S.G., Deeks J.J., Altman D.G. (2003). Measuring inconsistency in meta-analyses. BMJ.

[20] Dow C.T., Greenblatt C.L., Chan E.D., Dow J.F. (2022). Evaluation of BCG vaccination and plasma amyloid: a prospective, pilot study with implications for Alzheimer's disease. Microorganisms.

[21] Gofrit O.N., Klein B.Y., Cohen I.R., Ben-Hur T., Greenblatt C.L., Bercovier H. (2019). Bacillus Calmette-Guérin (BCG) therapy lowers the incidence of Alzheimer's disease in bladder cancer patients. PLoS One.

[22] Kim J.I., Zhu D., Barry E., Kovac E., Aboumohamed A., Agalliu I. (2021). Intravesical Bacillus calmette-Guérin treatment is inversely associated with the risk of developing Alzheimer disease or other dementia among patients with non-muscle-invasive bladder cancer. Clin. Genitourin. Cancer.

[23] Klinger D., Hill B.L., Barda N., Halperin E., Gofrit O.N., Greenblatt C.L. (2021). Bladder cancer immunotherapy by BCG is associated with a significantly reduced risk of Alzheimer's disease and Parkinson's disease. Vaccines.

[24] Makrakis D., Holt S.K., Bernick C., Grivas P., Gore J.L., Wright J.L. (2022). Intravesical BCG and incidence of Alzheimer disease in patients with bladder cancer: results from an administrative dataset. Alzheimer Dis. Assoc. Disord..

[25] Weinberg M.S., Zafar A., Magdamo C., Chung S.Y., Chou W.H., Nayan M. (2023). Association of BCG vaccine treatment with death and dementia in patients with non-muscle-invasive bladder cancer. JAMA Netw. Open.

[26] Cai Y., Liu J., Wang B., Sun M., Yang H. (2022). Microglia in the neuroinflammatory pathogenesis of Alzheimer's disease and related therapeutic targets. Front. Immunol..

[27] Hu D., Xia W., Weiner H.L. (2022). CD8(+) T cells in neurodegeneration: friend or foe?. Mol. Neurodegener..

[28] Zuo Z., Qi F., Yang J., Wang X., Wu Y., Wen Y. (2017). Immunization with Bacillus Calmette-Guérin (BCG) alleviates neuroinflammation and cognitive deficits in APP/PS1 mice via the recruitment of inflammation-resolving monocytes to the brain. Neurobiol. Dis..

[29] Schnier C., Janbek J., Lathe R., Haas J. (2022). Reduced dementia incidence after varicella zoster vaccination in Wales 2013–2020. Alzheimer’s Dement. Transl. Res. Clin. Interv..

